# Could computed tomography histogram analysis add value to the diagnosis of cholesteatoma?

**DOI:** 10.3906/sag-2105-12

**Published:** 2021-09-27

**Authors:** Nursel YURTTUTAN, Nagihan BİLAL, Betül KIZILDAĞ, Adem DOĞANER

**Affiliations:** 1 Department of Radiology, Faculty of Medicine, Kahramanmaraş Sütçü İmam University Kahramanmaraş, Turkey; 2 Department of Otorhinolaryngology, Faculty of Medicine, Kahramanmaraş Sütçü İmam University Kahramanmaraş, Turkey; 3 Department of Biostatistics and Medical Informatics, Faculty of Medicine, Kahramanmaraş Sütçü İmam University Kahramanmaraş, Turkey

**Keywords:** Histogram analysis, temporal bone, computed tomography, cholesteatoma

## Abstract

**Background/aim:**

To investigate the potential role of computed tomography (CT) histogram analysis in differentiating cholesteatoma (CHS) and non-cholesteatoma (NCHS).

**Materials and methods:**

We evaluated 77 temporal bone CT images (from November 2016 to February 2020) that were obtained pre-operatively (mean age, 37.10±17.27 years in CHS; 36.72±16.08 years in NCHS group). Histogram analyses of the resulting XML files were conducted using the R Project 3.3.2 program. ROC analysis was used to find threshold values, and the diagnostic efficiency of these values in differentiating CHS-NCHS was determined.

**Results:**

The CT images of 41 CHS (53.25%) and 36 NHCS cases (46.75%) were evaluated. There was a statistically significant difference between the CHS and NCHS group in terms of the mean, maximum, and median values (p = 0.036, p = 0.006, p = 0.043). When examining the ROC curve obtained from the mean of these parameters, area under the curve (AUC) is determined as 0.638, and when the threshold value is selected as 42.55, the mean value was determined to have a sensitivity of 86.50% and specificity of 56.10% in differentiating CHS-NCHS.

**Conclusion:**

In cases with magnetic resonance imaging (MRI) contraindications, small-sized lesions may be difficult to detect and characterize due to a poor resolution; to reduce the rate of false positives/negatives in these situations, CT histogram analysis of previously taken images may provide the additional information.

## 1. Introduction

Middle ear cholesteatoma (CHS) is a form of chronic otitis media requiring surgical intervention due to its severe complications [1]. A CHS diagnosis is usually made by an otorhinolaryngology physician through an otoscope examination. Preoperative imaging methods may be used to determine the extent of the disease, identify potential complications and tympanomastoid variations that present surgical risks, and verify the diagnosis in situations where otoscope examination is inconclusive. According to the guidelines of the GRADE Working Group, non-contrast high resolution computed tomography (HRCT) of the temporal bone is the primary choice for preoperative imaging of middle ear CHS [2, 3]. Magnetic resonance imaging (MRI) may be used to complement the findings of HRCT and also in certain indications.

In recent years, there has been a notable increase in research regarding computed tomography (CT) histogram analysis. Histogram analysis is a post-processing technique that is used to evaluate the intensity of the signals on digital images and their position relative to each other, and ultimately provides information by analyzing these differences through statistical software. This technique enables the quantification of several parameters within the region of interest (ROI), including mean, maximum, median, minimum, standard deviation (SD), variance, skewness, kurtosis, uniformity, entropy, etc. Thus, the distribution or relationship of gray pixel volume within the ROI can allow for an objective evaluation and interpretation and may provide additional information regarding the micro-environment of the tissue [4].

In this study, we aimed to comparatively analyze the histogram analysis measurements of temporal bone HRCTs that had been preoperatively and routinely taken from patients who have undergone surgery and have a surgical and histopathological diagnosis for CHS or non-cholesteatoma (NCHS) disease; we aimed to ultimately determine and report the accuracy of CT histogram analysis in predicting CHS preoperatively.

## 2. Materials and methods

### 2.1. Patient selection

The study was approved by the Kahramanmaraş Sütçü İmam University Institutional Local Ethics Board. Informed consent was not obtained as the data were collected retrospectively, and all imaging data were anonymized.

In this study, the researchers retrospectively scanned the hospital’s radiology information system (RIS) for cases with temporal bone HRCT images taken starting from February 2020 until reaching the sample size (November 2016). Using the hospital information system (HIS), the researchers recorded patients who had undergone a middle ear-mastoid surgery and had histopathological results. All patients in the case group received surgical intervention for the first time, and patients who had imaging taken before recurrent surgery were excluded from the study. A total of 77 patients who met the inclusion criteria were included. The patients were divided into two groups as the 41 CHS and 36 NCHS (including chronic granulation tissue and/or inflammation, cholesterol granuloma) cases.

### 2.2. Imaging technique

In this study, the standard non-contrast temporal bone HRCT images were taken with a 320 slice- Aquilion ONE (Toshiba Medical Systems, Otawara, Japan) scanner, and the acquisition parameters are as follows: tube voltage of 120 kVp, a tube current of 200 mAs, effective mAs 100, a slice thickness 0.5 mm, a slice interval 0.5 mm, a reconstruction increment 1 mm, a scan field of view (FOV) of 15–20 cm and a high-resolution matrix of 512 x 512. Coronal reformations were created through high-resolution axial isovolumetric data with a bone algorithm.

### 2.3. Imaging evaluation and analysis

**Figure 1:**
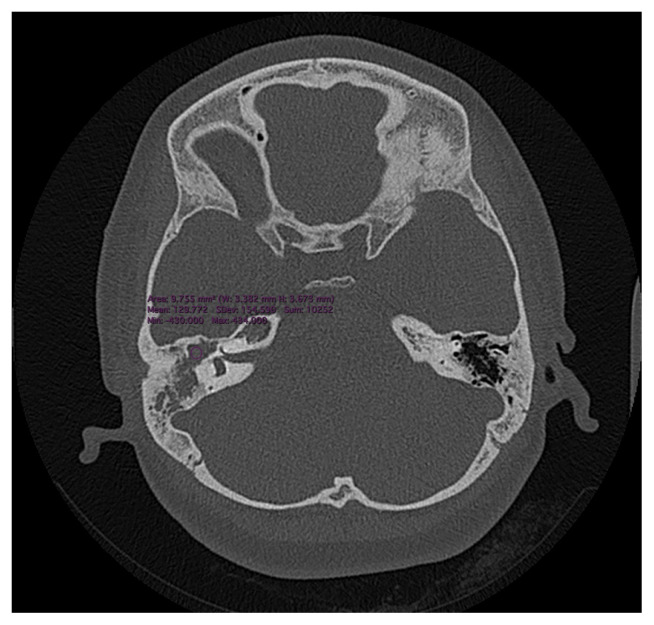
In the axial plane section of the 34 years old male cholesteatoma (CHS) patient’s non-contrast temporal bone high resolution computed tomography (HRCT), the region of interest (ROI) with pathological soft tissue density was hand-marked at the epitympanum level, and its transfer to an XML file is displayed.

**Figure 2:**
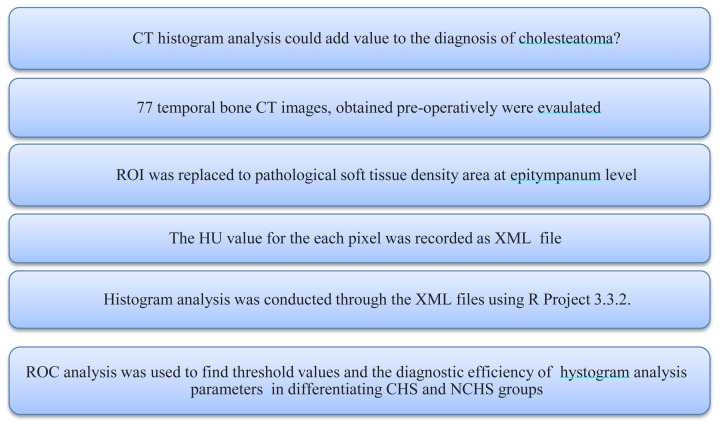
The diagram shows the basic concept of the study.

Images that met the criteria were evaluated with consensus by two experienced radiologists (11 and 17 years) on a workstation (27 inch iMac computer (Apple Inc. Cupertino, 88 California, USA)) through a blinded read. Considering a standard sized ROI (5–10 mm^2^), areas of pathological soft tissue density at the epitympanum level were hand-marked with a drawing tool, the Hounsfield Unit (HU) value of every pixel within the marked area was recorded to an XML (eXtensible Markum Language) file (Figure 1). In every case, a total of 30–60 (mean 47) pixels were worked out. Histogram analysis was conducted through the XML files using R Project 3.3.2. The histogram analysis included evaluation of mean, maximum, median, minimum, SD, variance, skewness, kurtosis, uniformity, and entropy parameters. ROC analysis was used to find threshold values, and the diagnostic efficiency of these values in differentiating CHS-NCHS was determined. The mean value is the average of a given set of data. The maximum parameter is the highest number expressed within the values of an analysis. The median value is defined as the value that divides an ordered ascending series of data into two from the middle. The diagram shows the basic concept of the study (Figure 2).

### 2.4. Statistical evaluation

Study data were evaluated using the R 3.3.2 program and IBM SPSS statistics, version 22 (IBM SPSS for Windows, version 22, IBM Corporation, Armonk, New York, United States). The normal distribution of data was evaluated using the Shapiro–Wilk test. For variables that did not exhibit normal distribution, the Mann–Whitney U test was used to compare the groups. The ROC curves were used to determine the sensitivity, specificity, and cut-off values. Statistical parameters were expressed as mean±SD and median (25% quartile-75% quartile). Statistical significance was expressed by a p value <0.05.

## 3. Results

A total of 77 temporal bone HRCT images were evaluated. The case group included 41 males and 36 females between the ages of 8 and 81 years (CHS group 37.10 ± 17.27, NCHS group 36.72 ± 16.08 years). Of the 41 CHS patients, 24 were male and 17 were female. Of the 36 NCHS patients, 17 were male and 19 were female (Table 1). There was no statistically significant difference between the groups in terms of age and gender (p = 0.922 and p = 0.321, respectively).

From the 10 parameters that were considered in the CT histogram analysis, there was a mild statistically significant difference between the CHS and NCHS group in terms of mean, maximum, and median values (in order p = 0.036, p = 0.006, and p = 0.043). Mean, maximum, and median values were statistically significantly higher in the CHS group compared to the NCHS group. Minimum, kurtozis, and uniformity values were higher in the CHS group, and SD, variance, skewness, and entropy values were higher in the NCHS group; however, these differences were not statistically significant (Table 2).

**Figure 3:**
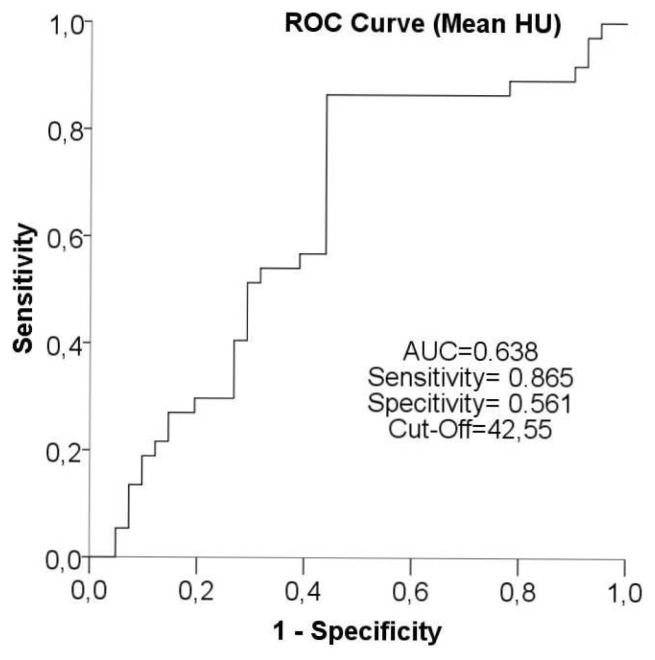
The ROC curve of the mean value, which is one of the computed tomography (CT) histogram analysis parameters used to differentiate cholesteatoma (CHS) and non-cholesteatoma (NCHS).

When examining the ROC curve obtained through the mean of statistically significant parameters in the histogram analysis, considering that the area under the curve (AUC) = 0.638 and when the threshold value is selected as 42.55, the mean value was determined to have a sensitivity of 86.50% and specificity of 56.10% in differentiating CHS-NCHS (Figure 3).

ROC analysis was conducted on the mean, maximum, and median parameters as they exhibited a statistically significant difference between the CHS and NCHS groups; with respect to the selected cut-off values, the resulting sensitivity and specificity values are displayed below (Table 3).

## 4. Discussion

In this study, statistically significant differences between the CHS and NCHS groups in terms of mean, maximum, and median values included in the CT histogram analysis parameters were detected. Mean, maximum, and median values were statistically significantly lower in the CHS group compared to the NCHS group. To the best of the researchers’ knowledge, this is the first study in the literature investigating the potential role of histogram analysis, a modern imaging method in radiology, in differentiating CHS -NCHS.

An objective and reliable imaging method is crucial for correctly identifying CHS prior to treatment and detecting postoperative residue or recurrence. Preoperative temporal bone HRCT has several advantages such as confirming the diagnosis, revealing the main complications, displaying the extent of the lesion, and contributing to surgical planning by showing the patient’s anatomy. For the surgeon, this contribution is especially significant when evaluating hidden areas such as the epitympanic recess and tympanic cavity [5]. The two primary findings of CHS on HRCT are a classic homogeneous non-calcified nodular tissue mass that is surrounded by areas of osteolysis [2]. In the early stages of the disease, the diagnosis of CHS may be difficult if bone changes are absent; the presence of dependant soft tissue or a mass loading effect on the ossicles are findings compatible with CHS [6]. An entirely full tympanic cavity makes it more difficult to distinguish CHS from correlated adjacent inflammatory reactions or granulation tissue [7, 8].

Canal wall up (CWU) and canal wall down (CWD) tympanoplasty are the primary techniques of CHS surgery. While CWU surgery has greater postoperative patient comfort, residual lesion rates are higher, and, because of this, second-look surgery is carried out after the 1st CWU tympanoplasty to assess the presence of and to treat residual lesions [9]. Residual-recurrence detection through otoscopic evaluation becomes increasingly difficult after tympanic membrane grafting, which makes postoperative imaging gain great importance [10]. Surgeons can safely postpone second-look surgeries if abnormal soft tissue is not detected in HRCT images taken 6–9 months after the initial surgery. CT holds a very high negative predictive value in the presence of an empty cavity after mastoidectomy [11, 12]. However, HRCT has a 43% sensitivity, 42%–51% specificity, and a 28% predictive value in detecting residual-recurrent CHS in the presence of soft tissue [11]. In this study, the question was whether we could increase the sensitivity and specificity of temporal bone CT examination.

The histogram of a structure is represented by numbers showing the specific gray value of the pixels within the structure. The distribution or relationship of gray pixel volume within the ROI can allow for an objective evaluation and interpretation and provide information regarding the micro-environment of the tissue. Using these values of the histogram, various parameters can be obtained such as mean, variance, and standard deviation [13]. Integrating this analysis with conventional imaging techniques can provide further detail regarding tissue nature. Recent studies have investigated the potential role of histogram analysis in the diagnosis and follow-up of tumor/tumor-like lesions as well as their benign-malign and aggressive-nonagressive differentiation [14, 15]. Alongside its use in oncology, there are also studies regarding the possible use of CT histogram analysis in showing liver and lung fibrosis as well as changes in the lens due to radiotherapy and also determining the changes in some anatomical regions of the brain in functional neurological disorders [15–19].

CHS is histo*p*athologically defined as an epidermoid cyst consisting of a lumen filled with desquamated epithelial debris and a subepithelial membrane affected by an inflammatory event containing cholesterol crystals and giant cells [20]. The lower mean, maximum, and median values found in the CHS group compared to the NCHS were consistent with histological content.

The ROC curve of the maximum was examined with a selected threshold value of 248.50, showing a sensitivity of 73% and specificity of 61% for differentiating CHS-NCHS. The ROC curve of the median was assessed with a selected threshold value of 50.25, revealing a sensitivity of 75.70% and specificity of 58.50% in differentiating CHS-NCHS. HRCT has a low sensitivity and specificity for detecting residual-recurrent CHS in the presence of soft tissue [11]; we believe this may be increased through the use of histogram analysis parameters.

In a CT study by Tok and colleagues, there was no statistically significant difference between CHS and COM in terms of soft tissue density and HU measurements [21]. When comparing studies, the higher number of patients in our study could have an impact on statistical significance.

In their studies regarding HU measurement in preoperative CT, Min-Hyun Park and colleagues reported a statistically significant difference between CHS and inflammatory granulation tissue as a result of measurements made at the mastoid antrum level. Being consistent with the results of our study, their mean HU value was found to be 42.68 ± 24.42 in the CHS group and 86.07 ± 26.50 in the NCHS group (our mean values were 39.6 in the CHS group and 65.71 in the NCHS group). They linked the low mean HU value in the CHS group to the destruction of trabecular bone structure in CHS patients [22].

Minimum, kurtozis, and uniformity median values were higher in the CHS group compared to the NCHS group, and SD, variance, skewness, and entropy values were higher in the NCHS group; however, these differences were not statistically significant.

Factors such as beam hardening, reconstruction artifact, scattered radiation, and material homogeneity could have an impact on HU values [23]. CT number can vary between machines, imaging techniques, and measurement methods. Therefore, the main purpose of our study is to investigate whether there is a difference between the parameters of both groups’ histogram analyses by conducting a detailed evaluation of the pixels through histogram analysis.

The use of DWI in postoperative patients could determine and alter the data of second look surgery or replace it altogether. However, prior to the initial operation, since temporal bone HRCT provides significantly higher resolution images compared to MRI, it is extremely valuable for confirming the patient’s diagnosis and identifying critical complications within anatomical features, and its ability to display fine anatomical detail is undoubtedly indispensable.

Nodular density findings (important clue for CHS) on HRCT cannot be considered in cases where the relative pockets of air are filled with soft tissue density. CT is insufficient in cases where bone erosion cannot be localized clearly, especially in areas hidden to the surgeon. Without requiring additional imaging of the patient and by installing a computer software, CT histogram analysis may be utilized to obtain detailed information regarding the inner structure of the aforementioned soft tissue density at the pixel level and contribute to the diagnosis. CT of the temporal bone may be preferred in postoperative follow-up imaging, especially in patients that cannot enter an MRI machine, those who require anesthesia for MRI, or to avoid the risks of possible contrast material exposure. In cases of false negative results in MRI where patients require noninvasive imaging prior to second-look surgery, additional information can be acquired from the histogram analysis of temporal bone HRCT.

Due to its high resolution, HRCT is favored in detecting small lesions and provides more information compared to MRI, and further data may be acquired through histogram analysis.

Our study included certain limitations; the number of patients in the study and control group were limited. The intra and inter-observer variability could not be evaluated as the measurements were made by consensus. The measurements were obtained manually and were made from the epithympanum-prusac distance, which may have induced bias in the evaluation of the images. Another limitation was that our study was conducted retrospectively. A limited number of pixels were worked out proportionally to the small ROI. To contribute to the literature, we believe that future studies should be conducted with patients that have undergone both DWI and temporal bone HRCT, where areas corresponding to restricted diffusion are confirmed through HRCT and subsequently measured and evaluated using histogram analysis.

In conclusion, because of a software that can be added to workstations and histogram analysis that can be utilized with ease in everyday practice, highly detailed data can be obtained through advanced pixel analysis, especially in debatable cases that require temporal bone CT examination or MRI sequences. Therefore, the repetitive use of additional imaging techniques, contrast materials, and radiation exposure could be avoided. In cases where MRI is contraindicated and the localization and high-resolution characterization of smaller lesions are difficult to obtain, histogram analysis of past images could be used to obtain greater amounts of data without additional imaging and can help avoid false-positive and negative situations.

## Figures and Tables

**Table 1:** Descriptive data of the study patients.

			Cholesteatoma (n = 41)	Non-cholesteatoma (n = 36)
Sex	Male	Right, n(%)	13(54.2)	7(41.2)
		Left, n(%)	11(45.8)	10(58.8)
	Female	Right, n(%)	14(82.4)	8(42.1)
		Left, n(%)	3(17.6)	11(57.9)
Age, Mean±SD			37.10 ± 17.27	36.72 ± 16.08

SD: Standard deviation

**Table 2:** Mean values of computed tomography histogram analyse parameters in cholesteatoma and non-cholesteatoma lesions

Parameters	Cholesteatoma (n=41)	Non-cholesteatoma (n=36)	
	Median(Q1–Q3)	Median(Q1–Q3)	p
Mean	39.61 (34.05–71.51)	**65.71 (48.09–80.46)**	**0.036** *
**Maximum**	**236.00 (198.00–302.00)**	**313.00 (246.00–373.00)**	**0.006** *
**Median**	**44.00(36.00–69.00)**	**70.00 (51.00–86.00)**	**0.043** *
Minimum	−149.00 (−234.00/−99.00)	−155.00 (−256.00/ −101.00)	0.791
SD	91.50 (68.28–110.45)	104.73 (74.42–132.93)	0.096
Variance	8372.57(4662–12198)	10967.66(5538.86–17671.10)	0.096
Skewness	−0.16 (−0.48−0.11)	−0.02 (−0.24−0.23)	0.074
Kurtosis	0.03 (−0.43−0.32)	−0.21 (−0.50−0.17)	0.337
Uniformity	0.25 (0.21–0.30)	0.22 (0.18–0.30)	0.248
Entropy	5.67 (5.34–6.03)	5.86 (5.52–6.03)	0.389

SD: Standard deviation

Mann–Whitney U test; α:0.05;

* Statistically significant.

**Table 3:** Diagnostic performance of texture analyses parameters for the diagnosis of cholesteatoma.

Parameters	AUC	p	Sensitivity	Specitivity	Cut-Off
Mean	0.638	0.036*	0.865	0.561	42.55
Maximum	0.681	0.006*	0.730	0.610	248.50
Median	0.633	0.043*	0.757	0.585	50.25

AUC: Area under curve.

Roc Curve- α:0.05;

* Statistically significant.

## References

[1] Ayache D Schmerber S Lavieille JP Roger G Gratacap B Middle ear cholesteatoma Ann Otolaryngol Chir Cervicofac 2006 123 3 120 137 (in French) 10.1016/s0003-438x(06)76653-1 16840901

[2] Ayache D Darrouzet V Dubrulle F Vincent C Bobin S French Society of Otolaryngology Head and Neck Surgery (SFORL) Imaging of non-operated cholesteatoma: clinical practice guidelines European Annals of Otorhinolaryngology Head and Neck Diseases 2012 129 3 148 152 10.1016/j.anorl.2011.09.005 22321912

[3] Atkins D Best D Briss PA Eccles M Falck-Ytter Y GRADE Working Group Grading quality of evidence and strength of recommendations British Medical Journal 2004 19 328 7454 1490 10.1136/bmj.328.7454.1490 15205295 PMC428525

[4] Castellano G Bonilha L Li LM Cendes F Texture analysis of medical images Clinical Radiology 2004 59 12 1061 1069 10.1016/j.crad.2004.07.008 15556588

[5] Gulati M Gupta S Prakash A Garg A Dixit R HRCT imaging of acquired cholesteatoma: a pictorial review Insights Imaging 2019 10 1 92 10.1186/s13244-019-0782-y 31578644 PMC6775179

[6] Juliano AF Ginat DT Moonis G Imaging review of the temporal bone: part I. Anatomy and inflammatory and neoplastic processes Radiology 2013 269 1 17 33 10.1148/radiol.13120733 24062560

[7] Banerjee A Flood LM Yates P Clifford K Computed tomography in suppurative ear disease: does it influence management? Journal of Laryngology & Otology 2003 117 6 454 458 10.1258/002221503321892280 12818053

[8] Alzoubi FQ Odat HA Al-Balas HA Saeed SR The role of preoperative CT scan in patients with chronic otitis media European Archives of Oto-Rhino-Laryngology 2009 266 6 807 809 10.1007/s00405-008-0814-6 18802717

[9] Gaillardin L Lescanne E Morinière S Cottier JP Robier A Residual cholesteatoma: prevalence and location. Follow-up strategy in adults European Annals of Otorhinolaryngology Head and Neck Diseases 2012 129 3 136 140 10.1016/j.anorl.2011.01.009 21955464

[10] Khemani S Singh A Lingam RK Kalan A Imaging of postoperative middle ear cholesteatoma Clinical Radiology 2011 66 8 760 767 10.1016/j.crad.2010.12.019 21524417

[11] Corrales CE Blevins NH Imaging for evaluation of cholesteatoma: current concepts and future directions Current Opinion in Otolaryngology & Head and Neck Surgery 2013 21 5 461 467 10.1097/MOO.0b013e328364b473 23880648

[12] Wake M Robinson JM Witcombe JB Bazerbachi S Stansbie JM Detection of recurrent cholesteatoma by computerized tomography after ‘closed cavity’ mastoid surgery Journal of Laryngology & Otology 1992 106 5 393 395 10.1017/s0022215100119644 1613362

[13] Miles KA Ganeshan B Hayball MP CT texture analysis using the filtration-histogram method: what do the measurements mean? Cancer Imaging 2013 13 3 400 406 10.1102/1470-7330.2013.9045 24061266 PMC3781643

[14] Ganeshan B Panayiotou E Burnand K Dizdarevic S Miles K Tumour heterogeneity in non-small cell lung carcinoma assessed by CT texture analysis: a potential marker of survival European Radiology 2012 22 4 796 802 10.1007/s00330-011-2319-8 22086561

[15] Kurtul N Yurttutan N Baykara M Investigation of the radiotherapy-related changes in the eye lens using computed tomography entropy analysis Journal of X-Ray Science and Technology 2018 26 5 747 755 10.3233/XST-18373 29889097

[16] Lubner MG Smith AD Sandrasegaran K Sahani DV Pickhardt PJ CT Texture Analysis: Definitions, Applications, Biologic Correlates, and Challenges Radiographics 2017 37 5 1483 1503 10.1148/rg.2017170056 28898189

[17] Park HJ Lee SM Song JW Lee SM Oh SY Texture-Based Automated Quantitative Assessment of Regional Patterns on Initial CT in Patients With Idiopathic Pulmonary Fibrosis: Relationship to Decline in Forced Vital Capacity American Journal of Roentgenology 2016 207 5 976 983 10.2214/AJR.16.16054 27533069

[18] Daginawala N Li B Buch K Yu H Tischler B Using texture analyses of contrast enhanced CT to assess hepatic fibrosis European Journal of Radiology 2016 85 3 511 517 10.1016/j.ejrad.2015.12.009 26860661

[19] Baykara S Baykara M Mermi O Yildirim H Atmaca M Magnetic resonance imaging histogram analysis of corpus callosum in a functional neurological disorder Turkish Journal of Medical Sciences 2021 51 1 140 147 10.3906/sag-2004-252 32892546 PMC7991863

[20] Valvassori Galdino E Mafee Mahmood F Carter Barbara L Imaging of the Head and Neck 2005 2nd ed Michigan University G Thieme Verlag 68 89

[21] Tok S Altınkaya N Ozer F Comparison Of Middle Ear Soft Tissue Density Of Chronic Otitis Media With Cholesteatoma By CT Ear Nose Throat Updates 2018 8 2 79 81 10.32448/entupdates.458964

[22] Park MH Rah YC Kim YH Kim JH Usefulness of computed tomography Hounsfield unit density in preoperative detection of cholesteatoma in mastoid ad antrum American Journal of Otolaryngology 2011 32 3 194 197 10.1016/j.amjoto.2010.01.008 20434800

[23] Levi C Gray JE McCullough EC Hattery RR The unreliability of CT numbers as absolute values American Journal of Roentgenology 1982 139 3 443 447 10.2214/ajr.139.3.443 6981306

